# Foresighting Outcomes and Risk Evaluation With Computational Artificial Intelligence in Stroke Trials: The FORECAST Study Protocol

**DOI:** 10.7759/cureus.96749

**Published:** 2025-11-13

**Authors:** Dipannita Adhikary, Adneen Moureen, Gie Ken-Dror, Md. Abdullah Yusuf, Redoy Ranjan, Pankaj Sharma

**Affiliations:** 1 Biological Sciences, Royal Holloway, University of London, London, GBR; 2 TB New Technologies and Diagnostics (Bangladesh), United States Agency for International Development (USAID), Dhaka, BGD; 3 Department of Microbiology, National Institute of Neurosciences & Hospital, Dhaka, BGD; 4 Cardiac Surgery, Bangabandhu Sheikh Mujib Medical University, Dhaka, BGD

**Keywords:** risk prediction model, stroke, stroke condition in bangladesh, stroke prognosis, stroke risk predictors

## Abstract

Stroke remains a leading cause of disability and mortality in Bangladesh, with limited data on risk stratification models tailored to the local population. Current prediction models inadequately capture the complex interplay of socioeconomic, environmental, and mental health factors prevalent in Bangladesh. This study protocol was designed to develop an artificial intelligence (AI)-based risk prediction model for stroke occurrence and poor long-term outcomes, specifically for the Bangladeshi population. The Foresighting Outcomes and Risk Evaluation with Computational Artificial Intelligence in Stroke Trials (FORECAST) study is an ambispective case-control study that will enroll 4,000 participants (2,000 stroke cases and 2,000 controls) from the National Institute of Neurosciences and Hospital, Bangladesh, between May 2022 and April 2026. We will develop ensemble machine learning models using comprehensive phenotypic data, including sociodemographic variables, comorbidities, anxiety/depression scores, and clinical parameters. The study will employ explainable AI techniques to enhance clinical interpretability and validation through k-fold cross-validation methods. The FORECAST study will establish the first AI-based stroke prediction model for Bangladesh, accounting for unique local risk factors, including rural-urban disparities and mental health comorbidities, with expected accuracy exceeding 90% based on recent advances in stroke prediction modeling. This study addresses critical gaps in stroke risk assessment for Bangladesh and similar low-middle-income countries by incorporating culturally relevant risk factors into advanced machine learning frameworks.

## Introduction

Stroke represents a significant global public health issue, and increasing incidence rates have substantially contributed to the national disease burden in Bangladesh [1]. The Global Burden of Disease Study 2019 identified stroke as a leading cause of death and disability-adjusted life years in South Asian populations, with Bangladesh experiencing particularly high stroke mortality rates, which may be attributed to an underdeveloped primary healthcare system and persistent financial constraints [2]. Current risk prediction models, predominantly developed in Western populations, inadequately capture the complex interplay of socioeconomic, environmental, and mental health factors prevalent in Bangladesh [3]. Recent advances in artificial intelligence (AI) have revolutionized stroke prediction capabilities, with ensemble learning methods achieving accuracies up to 99% and incorporating explainable AI techniques for enhanced clinical interpretability [4,5,6]. However, these sophisticated models remain largely untested in Bangladeshi populations, where unique risk factors, including high rural population density, limited healthcare access, and significant socioeconomic disparities, influence stroke outcomes [6]. Traditional risk assessment tools fail to account for these contextual factors, resulting in suboptimal predictions and missed opportunities for prevention [7].

Emerging evidence suggests that mental health comorbidities, particularly anxiety and depression, significantly influence stroke risk through complex pathophysiological mechanisms, including chronic inflammation, endothelial dysfunction, and behavioral factors [8,9]. Despite this growing recognition, the relationship between mental health and stroke remains underexplored in Bangladeshi populations, where cultural stigma and limited mental health resources create additional barriers to comprehensive care [10]. Furthermore, educational attainment and rural-urban residence patterns in Bangladesh create distinct risk profiles that require specialized modeling approaches to achieve optimal prediction accuracy [11].

The FORECAST (Foresighting Outcomes and Risk Evaluation with Computational Artificial Intelligence in Stroke Trials) study will address these critical knowledge gaps by developing and validating an AI-based risk prediction model specifically designed for Bangladeshi populations. By integrating advanced machine learning techniques with comprehensive phenotypic characterization, this study will establish a culturally appropriate and clinically actionable tool for stroke risk stratification in resource-limited settings.

## Materials and methods

The FORECAST study is an ongoing ambispective case-control study conducted at the National Institute of Neurosciences and Hospital (NINS&H), Bangladesh, in collaboration with the Institute of Cardiovascular Research, Royal Holloway, University of London, UK. This design allows for comprehensive characterization of stroke risk factors while maintaining feasibility within the Bangladeshi healthcare infrastructure between May 2022 and April 2026. The primary objective is to develop an AI-based risk prediction model that identifies independent predictors of stroke occurrence and poor long-term outcomes among Bangladeshi individuals. The study will also utilize explainable AI techniques to enhance the clinical interpretability of CT and MRI brain imaging findings, leveraging recent advances in stroke prediction modeling, with an anticipated accuracy rate exceeding 90%.

The study has received ethics approval from the institutional review boards of the National Institute of Neurosciences and Hospital (NINS&H), Dhaka, Bangladesh (reference number IRB/NINS/2025/492). Given the retrospective nature of case identification, a waiver of informed consent has been granted for historical data analysis, while prospective participants will provide written informed consent. Data anonymization follows international standards with secure storage and restricted access protocols. Written informed consent will also be obtained from a legal representative if the patient is unconscious or intubated.

Inclusion criteria encompass complete medical records availability and Bangladeshi residency for ≥5 years, while exclusion criteria include incomplete clinical data and previous stroke history for first-ever stroke analyses. Cases include adult patients (≥18 years) with acute ischemic or hemorrhagic stroke confirmed by a neuroimaging scan, either a CT/MRI scan, evaluated by two experts (neurologist and intervention radiologist). Stroke diagnosis will follow American Stroke Association (ASA) guidelines, confirmed by clinical evaluation and CT or MRI imaging. All patients with any form or severity of stroke, including mild, severe, prior, recurrent, critically ill, or intubated cases, will be included to reduce selection and reporting bias. Individuals with major comorbidities, such as other neurological disorders (e.g., brain tumors), severe systemic illnesses (e.g., cancer), secondary strokes due to other conditions (e.g., endocarditis, trauma), or those taking medications affecting stroke risk, will be excluded. Controls comprise age and sex-matched healthy adults without stroke history, recruited from hospital visitors and community health facilities, ensuring representative sampling across urban and rural populations. Controls will be 18 years or older, have no history of stroke, and must provide written informed consent. Hospitalized individuals are not eligible as controls. If spouse controls are unavailable, age- and sex-matched community volunteers will be recruited, with gender balance ensured through equal representation or sex stratification in analysis. The study will maintain equal proportions of males and females in both groups to address sex-related differences in stroke risk. Controls will be selected from the same geographic and socioeconomic backgrounds as cases to minimize population stratification and ensure comparability in environmental factors, lifestyle, and medication use.

The study will enroll 4,000 participants, comprising 2,000 stroke cases and 2,000 matched controls. Sample size calculations utilized established formulas for case-control studies with binary exposure, assuming 80% power, 5% significance level, and expected stroke prevalence of 35% in the exposed group versus 15% in the unexposed group [12,13]. This sample size ensures adequate power to detect clinically meaningful associations while accounting for potential data missingness and subgroup analyses. The formula for case-control studies with binary exposure:

n = [P₁(1-P₁) + P₀(1-P₀)] × (Zβ + Zα/2)² / (P₁ - P₀)²

where power (1-β): 80% (Zβ = 0.84); α: 5% (Zα/2 = 1.96), expected stroke prevalence in exposed group (P₁): 35%, expected stroke prevalence in unexposed group (P₀): 15%, and minimum detectable odds ratio: 2.0.

Calculation:

n = [0.35×0.65 + 0.15×0.85] × (0.84 + 1.96)² / (0.35 - 0.15)²

n = [0.2275 + 0.1275] × 7.84 / 0.04

n = 0.355 × 196 = 69.58 ≈ 1,740 per group

Accounting for 15% loss to follow-up and missing data: 1,740 × 1.15 = 2,000 per group

The total sample size will comprise 4,000 participants, divided into 2,000 cases and 2,000 controls.

Comprehensive data collection encompasses sociodemographic variables (age, sex, education level, rural-urban residence, occupation, income), clinical parameters (blood pressure, glucose levels, lipid profiles, body mass index), comorbidities (hypertension, diabetes mellitus, atrial fibrillation, coronary artery disease), lifestyle factors (smoking, alcohol consumption, physical activity), and mental health assessments using validated instruments including the Generalized Anxiety Disorder-7 (GAD-7) and Patient Health Questionnaire-9 (PHQ-9) scales [14,15]. Outcome measures include stroke occurrence (primary outcome) and long-term functional outcomes assessed using the Modified Rankin Scale at 90 days post-stroke (secondary outcome) [16]. Additional outcomes encompass stroke recurrence, mortality, and healthcare utilization patterns to provide comprehensive prognostic information.

The study will implement an ensemble machine learning approach integrating multiple algorithms to optimize prediction accuracy [17]. The modeling framework comprises four specialized modules: a sociodemographic module utilizing neural networks for processing education, rural-urban status, and socioeconomic factors; a clinical risk module employing random forest algorithms for traditional risk factors; a mental health module using gradient boosting to capture anxiety-depression patterns; and an outcome prediction module combining all inputs for final risk stratification [18,19].

Feature selection will employ correlation-based, mutual information-based, and wrapper-based methods to identify optimal predictor combinations while minimizing overfitting [20]. The study will address data imbalance issues through the Synthetic Minority Oversampling Technique (SMOTE) and other resampling methods to ensure robust model performance across all risk categories [21]. Model validation will utilize k-fold cross-validation (k = 10) with stratified sampling to ensure representative distribution across subgroups [22]. Performance evaluation will encompass accuracy, sensitivity, specificity, area under the receiver operating characteristic curve (AUC-ROC), and F1-score metrics, with target performance exceeding 90% accuracy based on recent advances in stroke prediction modeling [23,24].

**Figure 1:**
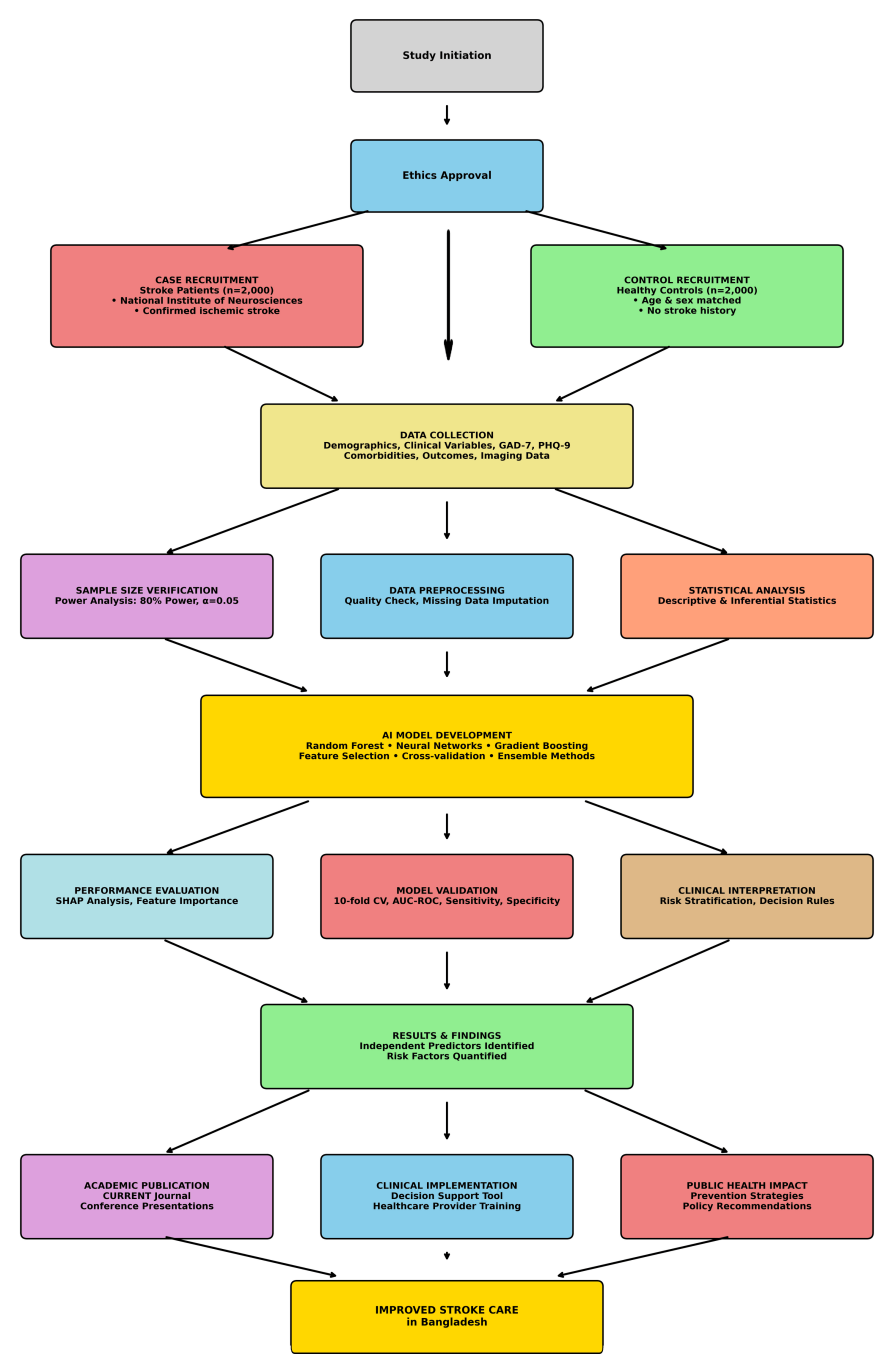
Dummy flowchart showing case-control recruitment to dissemination GAD: generalized anxiety disorder; PHQ: Patient Health Questionnaire; AI: artificial intelligence; AUROC: area under the receiver operating characteristic curve; mRS: Modified Rankin Scale Image created by the authors

To enhance clinical interpretability and trust, the study will integrate explainable AI techniques, including Shapley Additive Explanations (SHAP) and Local Interpretable Model-Agnostic Explanations (LIME) [25]. These methods will provide insights into feature importance, individual prediction explanations, and model decision-making processes, facilitating clinical adoption and regulatory approval (Figure 1).

Statistical analysis

Statistical analyses will employ appropriate methods for case-control studies, including conditional logistic regression for matched data and multiple imputation for missing values. Subgroup analyses will examine risk patterns across rural-urban populations, educational strata, and age groups to identify population-specific risk factors. Model calibration will be assessed using Hosmer-Lemeshow goodness-of-fit tests and calibration plots [26].

## Results

Expected results and clinical impact

**Figure 2:**
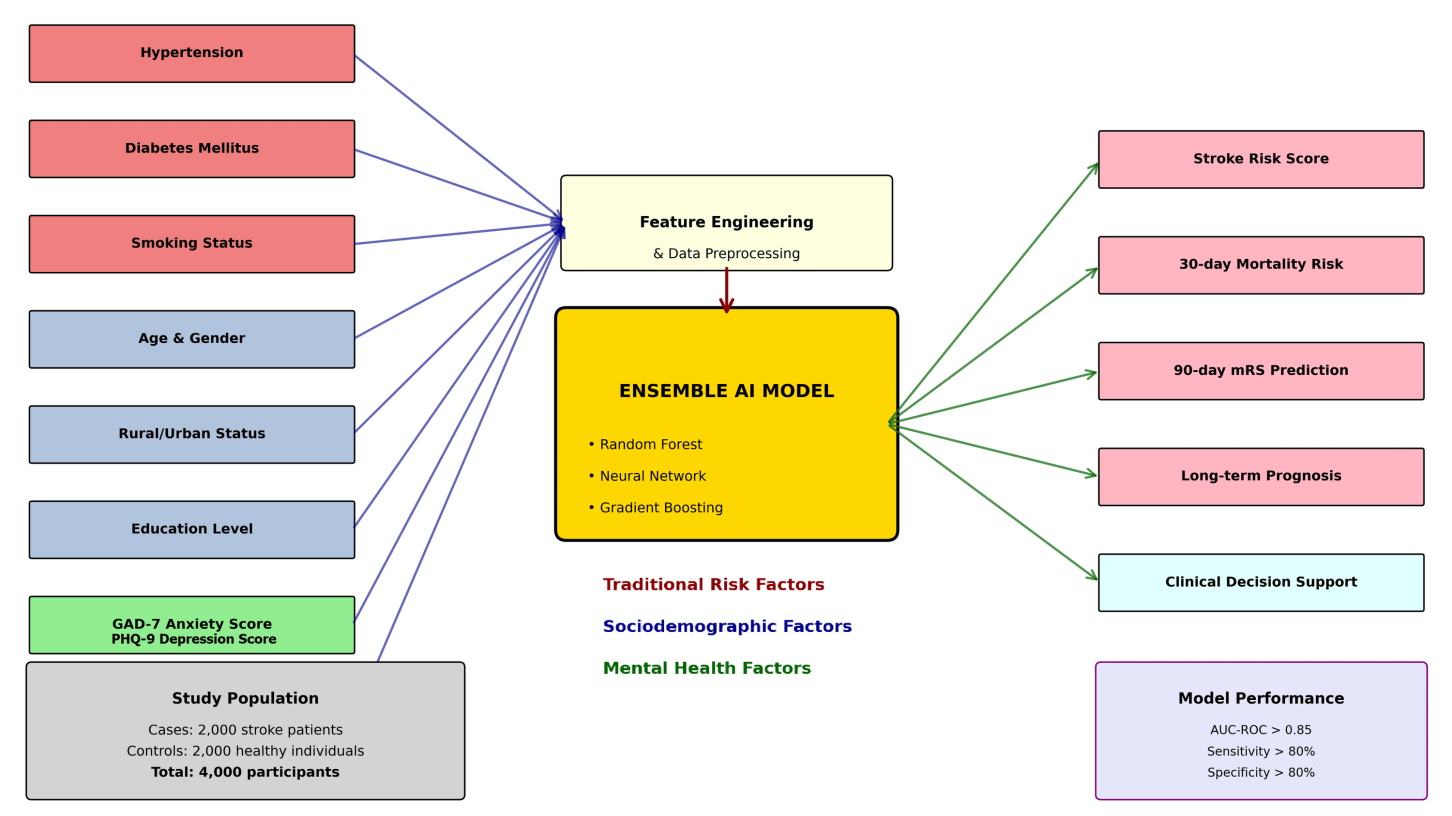
Dummy risk prediction model of the FORECAST study GAD: generalized anxiety disorder; PHQ: Patient Health Questionnaire; AI: artificial intelligence; AUROC: area under the receiver operating characteristic curve; mRS: Modified Rankin Scale; FORECAST: Foresighting Outcomes and Risk Evaluation with Computational Artificial Intelligence in Stroke Trials Image created by the authors

The FORECAST study will deliver the first AI-based stroke prediction model (Figure 2) specifically designed for Bangladeshi populations, with expected clinical and scientific impacts encompassing improved risk stratification leading to targeted prevention strategies, novel insights into mental health-stroke associations in South Asian populations, and cost-effective screening tools for resource-limited settings [27]. The model will provide personalized risk scores with confidence intervals, enabling clinicians to make evidence-based decisions regarding preventive interventions and resource allocation.

Population health benefits include reduced stroke burden through early identification and intervention, particularly in high-risk rural populations with limited healthcare access [28]. The study will also generate policy recommendations for stroke prevention programs and contribute to the development of mobile health applications for community-based screening.

## Discussion

The development of AI-based risk prediction models for stroke represents a paradigm shift in preventive neurology, offering unprecedented opportunities to enhance primary prevention strategies and improve patient outcomes [4,5]. Traditional stroke risk assessment tools, while valuable, demonstrate significant limitations in capturing the multifaceted nature of stroke pathogenesis, particularly in diverse populations with unique socioeconomic and cultural contexts [29]. The integration of AI technologies addresses these limitations by processing vast amounts of complex, interconnected data to identify subtle patterns and risk associations that conventional statistical methods may overlook.

The importance of developing population-specific prediction models cannot be overstated, particularly for Bangladesh, where conventional Western-derived risk calculators demonstrate poor calibration and discrimination [30]. The FORECAST study's focus on incorporating mental health variables represents an important advance, given emerging evidence linking depression and anxiety to increased stroke risk through mechanisms including chronic systemic inflammation, endothelial dysfunction, and poor medication adherence [29,30]. Furthermore, the inclusion of socioeconomic determinants such as rural-urban residence and educational attainment addresses critical social determinants of health that significantly influence stroke outcomes in resource-limited settings.

The clinical implications of accurate AI-based risk prediction extend beyond individual patient care to encompass population health strategies and healthcare resource allocation. By identifying high-risk individuals before stroke event occurrence, clinicians can implement targeted interventions, including intensive blood pressure management, anticoagulation therapy, lifestyle modifications, and mental health support, potentially preventing thousands of strokes annually [12,30]. Moreover, the explainable AI components of the FORECAST model will enhance clinical adoption by providing transparent, interpretable risk assessments that support shared decision-making between patients and healthcare providers, ultimately improving treatment adherence and outcomes. While the proposed study is promising, there are several limitations to consider. The sample size may not fully reflect the clinical and environmental diversity of the Bangladeshi population, which could affect how well the AI-based stroke risk prediction model applies to broader groups. The AI-based risk prediction model offers a powerful tool for identifying potential risk factors and predicting long-term poor prognosis among stroke patients in Bangladesh. Its strength lies in the ability to analyse large, complex datasets, uncovering subtle patterns that may not be apparent through traditional statistical methods. By integrating demographic, clinical, and imaging data, the model can provide personalised risk assessments to support early intervention and improve patient outcomes. However, limitations include dependence on data quality and completeness, as inconsistencies or biases in local healthcare records may reduce accuracy. In addition, the model’s generalisability may be constrained by limited external validation, making it essential to test and refine it across diverse populations and clinical settings within Bangladesh. Data will mostly come from selected hospitals, so rural or underserved populations may not be represented, especially where healthcare access and reporting differ. Further, the AI model’s accuracy depends on data quality, which could be affected by inconsistent diagnostic criteria, missing data, differences in imaging protocols, or variations in lab measurements. Moreover, without external validation using independent, multi-center groups, the model’s usefulness outside the study population is limited. The AI algorithm’s complex nature may also make it harder to interpret results for clinical decisions.

## Conclusions

The FORECAST study represents a significant advancement in stroke prediction modeling for Bangladesh, addressing critical gaps in current risk assessment tools through the integration of culturally relevant risk factors into sophisticated AI frameworks. By incorporating unique local determinants, including mental health comorbidities, socioeconomic factors, and rural-urban disparities, this study will enhance clinical decision-making and contribute substantially to stroke prevention efforts in Bangladesh and similar low-middle-income countries. The expected outcomes will establish a foundation for personalized stroke prevention strategies and inform future research directions in AI-based healthcare applications for underserved populations.

## References

[1] (2021). Global, regional, and national burden of stroke and its risk factors, 1990-2019: a systematic analysis for the Global Burden of Disease Study 2019. Lancet Neurol.

[2] Bangladesh Bureau of Statistics (2022). Bangladesh Bureau of Statistics. Population and Housing Census 2022: Preliminary Report. Population and Housing Census 2022: preliminary report.

[3] Mondal MB, Hasan AT, Khan N, Mohammad QD (2022). Prevalence and risk factors of stroke in Bangladesh: a nationwide population-based survey. eNeurologicalSci.

[4] Yangi K, Hong J, Gholami AS (2025). Deep learning in neurosurgery: a systematic literature review with a structured analysis of applications across subspecialties. Front Neurol.

[5] Yangi K, On TJ, Xu Y (2025). Artificial intelligence integration in surgery through hand and instrument tracking: a systematic literature review. Front Surg.

[6] Chowdhury MA, Uddin MJ, Khan HM, Haque MR (2015). Type 2 diabetes and its correlates among adults in Bangladesh: a population based study. BMC Public Health.

[7] Axford D, Sohel F, Abedi V (2024). Development and internal validation of machine learning-based models and external validation of existing risk scores for outcome prediction in patients with ischaemic stroke. Eur Heart J Digit Health.

[8] Byna A, Lakulu MM, Panessai IY, Nurhaeni N (2024). Machine learning-based stroke prediction: a critical analysis. Int J Adv Sci Eng Inf Technol.

[9] Rahman A, Bin F, Rahman A (2023). Early prediction of ischemic stroke using machine learning boosting algorithms. Proceedings of the 2023 3rd International Conference on Electrical, Computer, Communications and Mechatronics Engineering.

[10] Hossain S, Hasan MK, Faruk MO, Aktar N, Hossain R, Hossain K (2024). Machine learning approach for predicting cardiovascular disease in Bangladesh: evidence from a cross-sectional study in 2023. BMC Cardiovasc Disord.

[11] Zhang Y (2025). Stroke prediction based on machine learning. ITM Web Conf.

[12] Ranjan R, Adhikary D, Ken-Dror G, Yusuf MA, Moureen A, Hakim M, Sharma P (2024). Anthropometric measurements in predicting haemorrhagic stroke among Bangladeshi population: the MAGPIE study. J Multidiscip Healthc.

[13] Yusuf MA, Ranjan R, Adhikary D, Moureen A, Hakim M (2025). Association of paternal diabetes with hemorrhagic stroke in Bangladeshi women: the MAGPIE study. Health Sci Rep.

[14] Spitzer RL, Kroenke K, Williams JB, Löwe B (2006). A brief measure for assessing generalized anxiety disorder: the GAD-7. Arch Intern Med.

[15] Kroenke K, Spitzer RL, Williams JB (2001). The PHQ-9: validity of a brief depression severity measure. J Gen Intern Med.

[16] van Swieten JC, Koudstaal PJ, Visser MC, Schouten HJ, van Gijn J (1988). Interobserver agreement for the assessment of handicap in stroke patients. Stroke.

[17] Dritsas E, Trigka M (2022). Stroke risk prediction with machine learning techniques. Sensors (Basel).

[18] Dubey Y, Tarte Y, Talatule N, Damahe K, Palsodkar P, Fulzele P (2024). Explainable and interpretable model for the early detection of brain stroke using optimized boosting algorithms. Diagnostics (Basel).

[19] Moulaei K, Afshari L, Moulaei R, Sabet B, Mousavi SM, Afrash MR (2024). Explainable artificial intelligence for stroke prediction through comparison of deep learning and machine learning models. Sci Rep.

[20] Sirsat MS, Fermé E, Câmara J (2020). Machine learning for brain stroke: a review. J Stroke Cerebrovasc Dis.

[21] El-Geneedy M, El-Din Moustafa H, Khater H, Abd-Elsamee S, Gamel SA (2025). A comprehensive explainable AI approach for enhancing transparency and interpretability in stroke prediction. Sci Rep.

[22] Asadi F, Rahimi M, Daeechini AH, Paghe A (2024). The most efficient machine learning algorithms in stroke prediction: a systematic review. Health Sci Rep.

[23] Vu T, Kokubo Y, Inoue M (2024). Machine learning approaches for stroke risk prediction: findings from the suita study. J Cardiovasc Dev Dis.

[24] Rahman A, Hossain MS, Muhammad G (2022). Federated learning-based AI approaches in smart healthcare: concepts, taxonomies, challenges and open issues. Cluster Comput.

[25] Lundberg SM, Lee SI (2017). A unified approach to interpreting model predictions. Adv Neural Inf Process Syst.

[26] Hosmer DW, Lemeshow S (1980). Goodness of fit tests for the multiple logistic regression model. Commun Stat Theory Methods.

[27] Gatla TR (2024). Comparative evaluation of AI models for predicting stroke risk using genetic and lifestyle factors. Int J Innov Eng Res Technol.

[28] Klug J, Leclerc G, Dirren E, Carrera E (2024). Machine learning for early dynamic prediction of functional outcome after stroke. Commun Med (Lond).

[29] D'Agostino RB Sr, Vasan RS, Pencina MJ, Wolf PA, Cobain M, Massaro JM, Kannel WB (2008). General cardiovascular risk profile for use in primary care: the Framingham Heart Study. Circulation.

[30] Pandian JD, Gall SL, Kate MP (2018). Prevention of stroke: a global perspective. Lancet.

